# The effect of twin-to-twin delivery time intervals on neonatal outcome for second twins

**DOI:** 10.1186/s12884-018-1668-6

**Published:** 2018-01-19

**Authors:** L. Lindroos, A. Elfvin, L. Ladfors, U.-B. Wennerholm

**Affiliations:** 1 Department of Obstetrics and Gynecology, Institute of Clinical Sciences at Sahlgrenska Academy, Gothenburg University, Diagnosvägen 15, 416 85 Gothenburg, Sweden; 2 Department of Pediatrics, Institute of Clinical Sciences at Sahlgrenska Academy, Gothenburg University, Gothenburg, Sweden

**Keywords:** Second twin, Delivery time interval, Birth order, Cesarean section, Obstetric methods*, Neonatal, Outcome*, Pregnancy, Apgar score, Asphyxia Neonatorum

## Abstract

**Background:**

The objective was to examine the effect of twin-to-twin delivery intervals on neonatal outcome for second twins.

**Methods:**

This was a retrospective, hospital-based study, performed at a university teaching hospital in Western Sweden. Twin deliveries between 2008 and 2014 at ≥32 + 0 weeks of gestation, where the first twin was delivered vaginally, were included. Primary outcome was a composite outcome of metabolic acidosis, Apgar < 4 at 5 min or peri/neonatal mortality in the second twin. Secondary outcome was a composite outcome of neonatal morbidity.

**Results:**

A total of 527 twin deliveries were included. The median twin-to-twin delivery interval time was 19 min (range 2–399 min) and 68% of all second twins were delivered within 30 min. Primary outcome occurred in 2.6% of the second twins. Median twin-to-twin delivery interval was 34 min (8–78 min) for the second twin with a primary outcome, and 19 min (2–399 min) for the second twin with no primary outcome (*p* = 0.028). Second twins delivered within a twin-to-twin interval of 0–30 min had a higher pH in umbilical artery blood gas than those delivered after 30 min (pH 7.23 and pH 7.20, *p* <  0.0001). Secondary outcome was not associated with twin-to-twin delivery interval time. The combined vaginal-cesarean delivery rate was 6.6% (*n* = 35) and the rate was higher with twin-to-twin delivery interval >  30 min (*p* <  0.0001).

**Conclusions:**

An association, but not necessarily a causality, between twin-to-twin delivery interval and primary outcome was seen. An upper time limit on twin-to-twin delivery time intervals may be justified. However, the optimal time interval needs further studies.

## Background

Twin gestations are increasing worldwide as a result of higher maternal age and conceptions resulting from assisted reproductive technologies (ART) [1]. These pregnancies and deliveries are a challenge in obstetric practice. Twin gestations increase the risk of morbidity and mortality of both children, mainly due to preterm labor, intrauterine growth restriction and circumstances unique to twin pregnancies such as twin-twin transfusion syndrome (TTTS) and umbilical cord complications [2–4].

It is not only the pregnancy itself but also the delivery that constitutes a greater risk to twins than singletons. Kiely showed that normal sized twins (birthweight > 3000 g) had a 70% increased risk of perinatal mortality and a threefold increased risk of intrapartum death compared to singletons [5]. The second twin is at higher risk than the first twin [6, 7] mainly due to the second twin being more difficult to monitor and also because complications such as cord prolapse, premature placental separation and fetal distress during labor are more common in the second twin than in the first one [8–10].

Several studies over the years have resulted in quite a clear understanding of how to deliver twins with regard to their presentation and gestation. Vaginal delivery is advocated if the first twin is in cephalic presentation, both twins are normal sized (1500–4000 g) and the second twin is not substantially larger than the first [7]. There is however no consensus as to whether the time between deliveries of the first twin and the second twin affects the neonatal outcome for the second twin. Some studies show that the risks mentioned above increase with a prolonged twin-to-twin time interval >  30 min [9, 11] as well as the risk of a combined vaginal-cesarean delivery [3, 4]. A correlation between longer twin-to-twin delivery intervals and decreasing pH in umbilical arterial blood gas, as well as a reduction of Apgar scores in the second twin, have been found in several previous studies [11, 12]. It has been suggested that the time interval should be kept short, ideally below 30 min. However, there are also a few studies showing that this is not of clinical significance and that there is no need for an upper time limit [9, 13, 14]. Recent guidelines as those from The American College of Obstetricians and Gynecologists offer no guideline regarding the optimal delivery time interval [15].

The aim of the current study was to investigate the relation between twin-to-twin time interval and the neonatal outcome for the second twin, considering both metabolic acidosis and Apgar score at birth, as well as neonatal mortality and morbidity within the first 28 days of life. The study was conducted at an obstetric unit where active management of the delivery of the second twin is not advocated.

## Methods

After ethical approval from the Ethical committee at University of Gothenburg, Sweden, information regarding all twin deliveries at Sahlgrenska University Hospital, Gothenburg, Sweden, between 2008 and 2014 was retrieved from Obstetrix, a computerized obstetric medical record system. This university teaching hospital is the largest obstetric unit in Sweden and has an average annual delivery rate of 10,000 deliveries. A total of 527 twin deliveries were studied after exclusion of the following deliveries:

- delivery before 32 weeks of gestation
- the first twin delivered by cesarean section (CS)
- intrauterine death of one or both twins before onset of labor
- known fetal malformations or chromosome aberrations in one or both twins
- monoamniotic twin gestations

Because of its possible impact on neonatal outcome, information regarding maternal age, body mass index (BMI), smoking, parity, previous CS, maternal chronic hypertension, gestational hypertension, preeclampsia, diabetes mellitus type 1 or 2, gestational diabetes, gestational age, chorionicity, induction of labor, regional anesthesia, presentation, mode of delivery, time of delivery of both twins and birthweight was collected. From this information twin-to-twin time interval, large for gestational age (LGA), small for gestational age (SGA) and inter-twin birthweight discordance was calculated. LGA and SGA was defined as a birthweight of greater than + 2 standard deviations (SD) or less than − 2 SD, respectively, according to the Swedish reference value for singletons, with adjustment for gestational age and sex [16]. We used the reference values for singletons since there are no reference values available for twins. Inter-twin birthweight discordance was calculated as 100 × (birthweight of the largest twin minus birthweight of the smallest twin)/ birthweight of the largest twin, and was defined as a difference ≥ 25% [17]. Chorionicity was determined by ultrasonography in the first or second trimester.

Information regarding umbilical blood gas analysis including pH and base excess (BE), Apgar score, perinatal mortality and morbidity was retrieved from the obstetric computerized medical records system as well as from the Swedish neonatal quality register (SNQ), a national perinatal computerized medical record system in which all perinatal units in Sweden register their patients. After this it was possible to link the information from Obstetrix and SNQ to each other by means of the mother’s 10-digit social security number.

The twin-to-twin time interval was defined and calculated as the time interval between the delivery of the first twin and the second twin. In order to examine trends of pH, Apgar and neonatal outcome over time, the time interval was divided into periods of 15 min (1–15, 16–30, 31–45, 46–60 and > 60 min). Analyses of twin-to-twin time intervals < 30 min and > 30 min were also performed since some previous studies suggested an upper time limit of 30 min.

Gestational age was based on a routine ultrasound examination, usually made in the second trimester by an expert midwife or obstetrician in ultrasound. Gestational age is calculated in days but presented in weeks + days as this is the most common and generally accepted method.

Primary outcome was defined as a composite measure consisting of any of the following factors:

- severe metabolic acidosis in umbilical arterial blood gas with pH < 7.05 and BE < − 12 or pH < 7.00
- if no blood gas was available; Apgar < 4 at 5 min
- perinatal mortality defined as death after onset of labor and within 7 days after birth
- neonatal mortality as death within 28 days after birth

Secondary outcome was defined as a composite measure consisting of any of the following factors:

- Apgar < 7 at 5 min
- acidosis in umbilical arterial blood gas with pH < 7.10
- interventricular hemorrhage (IVH) verified by ultrasonography and/or magnetic resonance imaging (MRI) within 28 days after birth
- hypoxic ischemic encephalopathy I-III (HIE)
- seizure within 7 days after birth
- septicemia confirmed by positive blood culture within 28 days after birth
- necrotizing enterocolitis (NEC) within 28 days after birth
- infant respiratory distress syndrome (IRDS) and transient tachypnea of the newborn (TTN)
- need for assisted ventilation – continuous positive air pressure (CPAP), ventilation by mask or mechanical ventilation
- If gestational age ≥ 34 weeks; admittance to neonatal intensive care unit (NICU) > 7 days

The intrapartum management of all twin deliveries followed the department’s protocol. All twin pregnancies with the first twin in cephalic presentation were planned for vaginal delivery. Regional anesthesia was not mandatory. Continuous cardiotocography (CTG) was applied during active labor, predominately with fetal scalp registration on the first twin and external transducer for the second twin. A specialist in obstetrics and often resident in training as well as two midwives, at least one with experience, were present at delivery. If oxytocin was used it was paused immediately after the delivery of the first twin. Fetal presentation and heartrate of the second twin was examined with digital examination, ultrasonography and CTG. Amniotomy could be performed to enhance contractions if the second twin was engaged. If the second twin was in transverse lie an attempt of external version, to either cephalic or breech presentation, was performed - if needed with intravenous terbutaline or nitroglycerin for uterus relaxation. If lack of effective spontaneous contractions, oxytocin was started and spontaneous vaginal delivery, in head or breech presentation, was preferred. If this failed an internal version and breech extraction were to be considered. Otherwise CS was performed. There was no upper time limit for twin-to-twin time interval and if no complications occurred expectant management was applied. All twin deliveries were conducted at a special delivery ward which has an operating theatre where the CS were performed.

### Statistical analysis

For comparison between two groups independent T-tests were used for continuous variables with normal distributions, Mann-Whitney U-test for continuous variables with non-normal distributions and Fisher’s exact test for dichotomous variables. Logistic regression with calculation of odds ratio (OR) with 95% confidence interval (CI) and adjustment for gestational age was performed. Subgroup analysis was performed for chorionicity, presentation of the second twin and for twin pregnancies with vaginal deliveries of the second twin excluding cesarean deliveries of the second twin. Spearman’s rho was used for analysis of correlation between umbilical cord arterial pH and twin-to-twin delivery time interval. Statistical calculations were performed using SPSS Statistics 22. All significance tests were two-sided and conducted at the 5% significance level.

## Results

A total of 527 twin deliveries (1054 infants) met the inclusion criteria between January 2008 and December 2014. During this time period there were 71,908 deliveries at the obstetric unit with 1.6% (1123/71908) being twin deliveries between 22 + 1 to 41 + 2 weeks of gestation and with 23.9% (268/1123) elective CS before onset of labour.

The baseline maternal characteristics are described in Table 1. There were 501 twin gestations with known chorionicity, 386 (77%) were dichorionic and 115 (23%) were monochorionic.

**Table 1:** Baseline characteristics in women with twin deliveries after 32 completed gestational weeks and with vaginal delivery of the first twin

	Twin deliveries *N* = 527
Maternal age, years, mean (SD) median (range)	32.5 (4.8), 32.7 (17.5–51.4)
≥35 years	145 (27.5)
Nulliparous	202 (38.3)
Previous cesarean section	22 (4.2)
Body Mass Index, mean (SD) median (range)	24.2 (4.2) 23.4 (16–43) *n* = 452
Body Mass Index ≥35^a^	13/452 (2.8)
Smoking at first antenatal visit^a^	20/436 (4.6)
Chronic hypertension	2 (0.4)
Gestational hypertension	2 (0.4)
Preeclampsia	46 (8.7)
Diabetes mellitus type 1 or 2	1 (0.2)
Gestational diabetes mellitus	6 (1.1)
Dichoriotic/diamniotic twin gestations^b^	386/501 (77.0)
Monochoriotic/diamniotic twin gestations^b^	115/501 (23.0)
Pregnancies with twin-twin transfusion syndrome	2 (0.4)
Mode of delivery of the second twin	
Spontaneous cephalic	288 (54.6)
Spontaneous breech	123 (23.3)
Breech extraction	9 (1.7)
Vacuum extraction	72 (13.7)
Cesarean section	35 (6.6)

Values are n (%), unless otherwise stated

^a^There were missing values on Body Mass Index, smoking

^b^Chorionicity was unknown in 26 deliveries

In 463 (87.9%) deliveries the first twin had a spontaneous delivery in cephalic presentation, 61 (11.6%) had an assisted vaginal delivery (vacuum extraction/forceps) and although CS is advocated when the first twin is in breech there were three (0.6%) spontaneous vaginal breech deliveries. A total of 35 (6.6%) of the deliveries were combined vaginal/cesarean deliveries. In 411 (78.0%) deliveries the second twin had a spontaneous delivery in cephalic or breech presentation, in 72 (13.7%) an assisted vaginal delivery and in nine cases (1.7%) a breech extraction was performed.

Neonatal characteristics for the first and second twins are shown in Table 2. The median gestational age was 262 days (range 225–286 days) with 200/527 (38.0%) of the deliveries being preterm.

**Table 2:** Neonatal characteristics in the first and second twins in twin deliveries after 32 completed gestational weeks and with vaginal delivery of the first twin

	First twin	Second twin	*p*-value
	N = 527	N = 527	
Gestational age, days, mean (SD), median (range)	262 (13.7) 262 (225–286)		
≥ 37 + 0 weeks	327 (62)		
34 + 0–36 + 6 weeks	168 (31.9)		
32 + 0–33 + 6 weeks	32 (6.1)		
Fetal sex			
Female	263 (49.9)	260 (49.3)	0.90
Male	264 (50.1)	267 (50.7)	
Birthweight			
Birthweight, g, mean, (SD), median (range)	2695 (488) 2723 (1285–4350)	2673 (496) 2690 (1230–4065)	0.46
< 2500 g	187 (35.5)	193 (36.6)	0.75
< 1500 g	3 (0.6)	3 (0.6)	
SGA (< −21%)	113 (21.4)	140 (26.6)	0.06
LGA (> + 21%)	2 (0.4)	1 (0.2)	
Weight difference, %, mean (SD), median (range)	−12.1 (11.1) − 12.2 (− 54.4 − + 25.6)	− 12.8 (11.9) − 12.5 (− 56 − + 26.8)	0.31
Inter-twin birthweight discordance ≥25%	27 (5.1)		
First twin larger than second twin	16/27 (59.3)		

Values are n (%), unless otherwise stated

*SGA* Small for Gestational Age, *LGA* Large for Gestational Age, *SD* standard deviation

The median twin-to-twin time interval was 19 min (range 2–399) with 217 (41.2%) of the second twins being born within 15 min, 143 (27.1%) second twins were born within 16–30 min, 70 (13.3%) within 31–45 min, 37 (7%) within 46–60 min and 60 (11.4%) after 60 min.

Composite primary outcome occurred in 2.7% (14/527) in the second twins (Table 3). Of the 14 s twins with a composite primary outcome there were two cases of perinatal mortality. One was a case of intrapartum death after breech extraction at 34 weeks of gestation, where the obstetrician was unable to deliver the head (twin-to-twin time interval 29 min) and one was a second twin born at 33 weeks of gestation (twin-to-twin time interval eight minutes) who developed NEC at four days of age, underwent major surgery and died shortly thereafter. The remaining 12 s twins with composite primary outcomes had severe metabolic acidosis. One of these neonates, born at 38 weeks of gestation, was admitted to NICU for five days because of transient tachypnea of the newborn (TTN).The other 11 recovered within 15 min.

**Table 3:** Neonatal morbidity in first and second twins in twin deliveries after 32 completed gestational weeks and with vaginal delivery of the first twin

	First twin N = 527	Second twin N = 527	*p*-value
Composite primary outcome^a^	4 (0.7)	14 (2.7)	0.029
Apgar score < 4, 5 min^b^	0	2 (0.4)	1.0
pH < 7.05 and BE< −12 or pH < 7.00^c^	4 (1.0)	12 (2.8)	0.08
Perinatal mortality < 7 days	0	2 (0.4)	0.50
Neonatal mortality < 28 days	0	0	1.0
Composite secondary outcome^a^	68 (12.9)	92 (17.4)	0.048
Apgar score < 7, 5 min	4 (0.8)	9 (1.7)	0.46
pH < 7.10	5 (1.2)	40 (9.4)	< 0.0001
Admitted to NICU	129 (24.5)	123 (23.3)	0.72
NICU, mean (SD), median, (range) days	11 (7.3) 10 (1–35)	11 (7.0) 10 (1–37)	1.0
NICU > 7 days	81 (15.4)	76 (14.4)	0.90
IRDS	1 (0.2)	5 (0.9)	0.23
TTN	8 (1.5)	10 (1.9)	0.81
IVH	0	0	1.0
Septicemia	3 (0.6)	2 (0.4)	1.0
NEC	0	1 (0.2)	1.0
Convulsions	0	0	1.0
HIE	0	0	1.0
Assisted ventilation	26 (4.9)	30 (5.7)	0.68

Values are n (%), unless otherwise stated

*BE* Base Excess, *NICU* Neonatal Intensive Care Unit, *IRDS* Infant Respiratory Distress Syndrome, *TTN* Transient Tachypnea of the Newborn, *IVH* Intraventricular Hemorrhage, *NEC* Necrotizing Enterocolitis, *HIE* Hypoxic Ischemic Encephalopathy

^a^as defined in Material and Methods

^b^There were missing Apgar score in first twin (n = 5) and second twin (n = 3)

^c^There was missing pH values in first twin (*n* = 121) and second twin (*n* = 101)

There were two pregnancies diagnosed with TTTS included in the study. They delivered at 35 + 5 and 37 + 0 weeks of gestation with twin-to-twin time interval of 14 min and 11 min, respectively. The twin delivered at 35 + 5 weeks of gestations was admitted to NICU for 7 days because of suspected infection and seizures, both of which could not be confirmed. No other complications occurred.

Twin-to-twin time interval had a significant impact on the composite primary outcome for the second twin (Table 4). Median twin-to-twin time interval was 34 (8–78) min for the second twin with a composite primary outcome and 19 (2–399) min for the second twin without a composite primary outcome (*p* = 0.028). Lower mean birth weight was associated with a higher rate of primary outcome (*p* = 0.028) (Table 4).

**Table 4:** Composite primary (*n* = 14) and secondary outcome ^a^ (*n* = 92) in the second twin (*n* = 527) in relation to birth characteristics

	Primary outcome *N* = 14	No primary outcome *N* = 513	Primary outcome vs no primary outcome *p*-value	Secondary outcome *N* = 92	No secondary outcome *N* = 435	Secondary outcome vs no secondary outcome *p*-value
Twin-to-twin time interval, min, mean (SD) median (range)	37 (21) 34 (8–78)	30 (36) 19 (2–399)	0.028	37 (49) 21 (3–331)	29 (31) 18 (2–399)	0.13
Gestational age, days, mean (SD) median (range)	256 (14) 257 (226–279)	262 (13.7) 263 (225–286)	0.13	252 (14.1) 249 (226–281)	264 (12.6) 265 (225–286)	< 0.0001
Birthweight, g, mean (SD) median (range)	2384 (462) 2515 (1230–2905)	2680 (495) 2690 (1240–4065)	0.028	2365 (523) 2325 (1560–4050)	2737 (466) 2750 (1230–4065)	< 0.0001
Birthweight difference^b^ mean (SD) median (range)	−17.7 (14.7) − 17.4 (− 40.1–4)	− 12.7 (11.8) − 12.5 (− 56–26.8)	0.12	−14.9 (13.3) − 15.1 (− 46.8–26.8)	−12.4 (11.6) − 12.2 (− 56–20.8)	0.07
Small for gestational age (<− 21%)	6 (42.9)	134 (25.5)	0.23	29 (31.5)	111 (25.6)	0.24
Inter-twin birthweight discordance ≥25%	1 (7.1)	26 (5.1)	0.53	9 (9.8)	18 (4.1)	0.036
Monochoriotic twins	1 (7.1)	26 (5.1)	0.53	20 (21.7)	95 (21)	1.0
Assisted vaginal delivery	2 (14.2)	70 (13.7)	1.0	13 (13.5)	59 (13.7)	0.87
Spontaneous breech delivery	7 (50.0)	125 (24.4)	0.053	25 (27.2)	107 (24.7)	0.60
Breech extraction, including internal version	1 (7.1)	8 (1.6)	0.22	3 (3.3)	6 (1.4)	0.20
Cesarean section, emergency	2 (14.3)	20 (3.9)	0.11	6 (6.5)	16 (3.7)	0.25
Cesarean section, urgent^c^	0	13 (2.5)	1.0	5 (5.4)	8 (1.8)	0.06

Values are n (%) unless otherwise stated

^a^as defined in Methods

^b^according to Marsal et al. [16]

^c^urgent cesarean section is an emergency cesarean section performed without any delay and in general anesthesia without routine antiseptic cleaning

The composite secondary outcome occurred in 17.4% (92/527) in the second twin. This was mainly due to pH < 7.10 or the need for assisted ventilation (Table 3).

Median twin-to-twin time interval, 21 (3–331) min vs. 18 (2–399) min (*p* = 0.13), had no significant impact on the composite secondary outcome for the second twin. Factors associated with secondary outcomes were lower mean birthweight (*p* <  0.0001), lower gestational age (*p* <  0.0001) and inter-twin birthweight discordance ≥25% (*p* = 0.036) (Table 4).

**Fig. 1:**
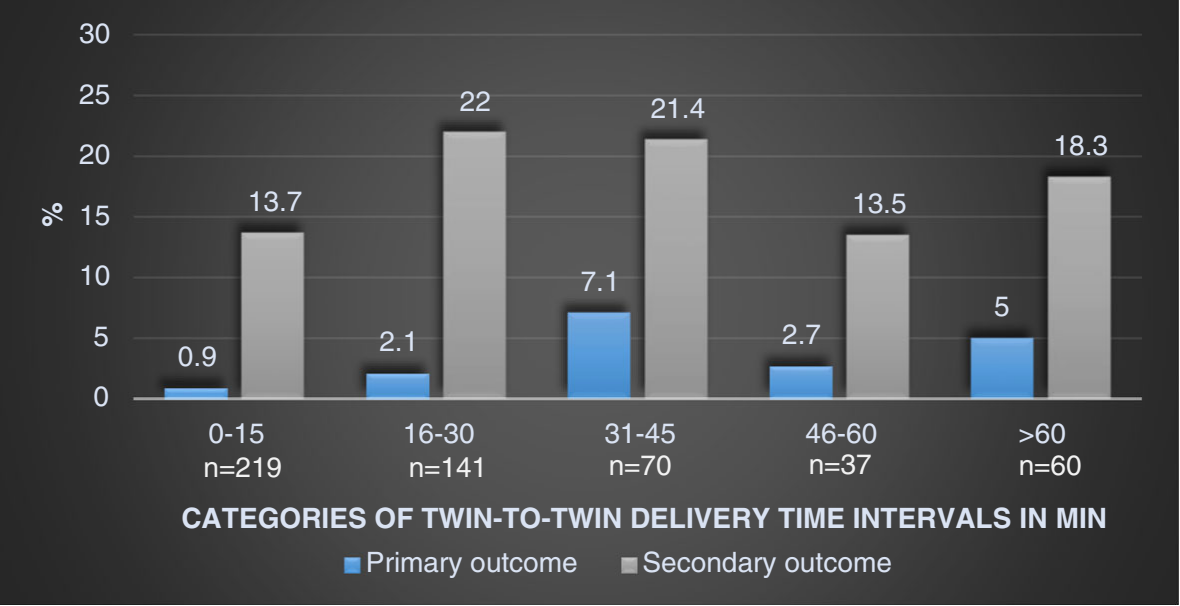
Composite primary* and secondary** outcome in the second twin according to twin-to-twin delivery time interval in minutes

Composite primary and secondary outcomes are shown in relation to the stratified twin-to-twin time intervals in Fig. 1.

When twin-to-twin time intervals were divided into groups of 0 to 30 min and > 30 min, a significant difference could be seen in composite primary outcome (*p* = 0.016), severe metabolic acidosis (*p* = 0.002), mean arterial (*p* < 0.0001) and mean venous umbilical cord pH (*p* = 0.016). The outcome was more adverse when the second twin was delivered > 30 min after the first twin (Table 5). The odds ratio (OR) for a composite primary outcome for the second twin delivered > 30 min vs ≤ 30 min was 4.0 (95% CI 1.3–12.2) and 4.1 (95% CI 1.4–12.6) after adjustment for gestational age. Also, when restricting the analysis to twins with a vaginal delivery (*n* = 492) a higher rate of the composite primary outcome occurred in twins delivered > 30 min after the first twin (*p* = 0.007), OR 5.3 (95% CI 1.6–17.9) and OR 5.6 (95% CI 1.6–18.9) after adjustment for gestational age.

**Table 5:** Composite primary and secondary outcomes* and mean umbilical cord arterial and venous pH values in the second twin according to twin-to-twin time interval ≤ 30 min or > 30 min

	0–30 min *N* = 360	> 30 min *N* = 167	*p*-value
Primary composite outcome	5 (1.4)	9 (5.4)	0.016
pH < 7.05 and BE < −12 or pH < 7.00	3/290 (1)	9/136 (6.6)	0.002
Apgar score < 4 at 5 min	2/357 (0.6)	0	1.0
Perinatal mortality	2 (0.6)	0	1.0
Neonatal mortality	0	0	1.0
Secondary composite outcome	61 (16.9)	31 (18.6)	0.71
Apgar < 7 at 5 min	8 (2.2)	1 (0.6)	0.28
pH < 7.10	18/290	22/136	0.002
Admitted to NICU	88/360	35/167	0.44
NICU, mean (SD), median, (range) days	11 (7.2) 10 (1–35)	12 (7.7) 9 (2–37)	0.26
NICU > 7 days	54/88	22/35	1.0
IRDS	4/360	1/167	1.0
TTN	6/360	4/167	0.73
Septicemia	2/360	0/167	1.0
NEC	1/360	0/167	1.0
Assisted ventilation	21/360	9/167	1.0
Mean a-pH (SD)	7.23 (0.08) *N* = 290	7.20 (0.09) *N* = 136	< 0.0001
Mean v-pH (SD)	7.29 (0.08) *N* = 284	7.27 (0.09) *N* = 140	0.016

Values are n (%) unless otherwise stated

*BE* Base Excess, *NICU* Neonatal Intensive Care Unit, *IRDS* Infant Respiratory Distress Syndrome, *TTN* Transient Tachypnea of the Newborn, *NEC* Necrotizing Enterocolitis, *a-pH* umbilical cord arterial pH, *v-pH* umbilical cord venous pH, *BE* Base Excess, *SD* standard deviation

^a^as defined in Methods

Stratified into chorionicity, the composite primary outcome occurred in 0/87 monochorionic twins delivered within 0 to 30 min and in 3.6% (1/28) delivered > 30 min after the first twin (*p* = 0.243). Composite secondary outcome occurred in 16.1% (14/87) and 21.4% (6/28) monochorionic twins delivered within 0–30 min and > 30 min after the first twin, respectively (*p* = 0.569). The composite primary outcome occurred in 1.6% (4/256) and 6.2% (8/130) dichorionic twins delivered within 0–30 min and > 30 min after the first twin, respectively (*p* = 0.025). There was no significant difference in composite secondary outcome for dichorionic twins delivered within 30 min or > 30 min after the first twin.

There was no difference in composite primary or secondary outcomes according to presentation (cephalic or breech) and intertwin delivery interval.

**Fig. 2:**
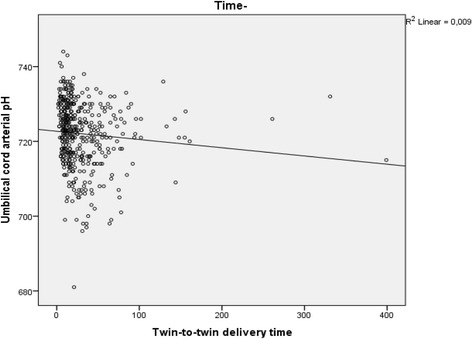
Umbilical cord arterial pH in the second twin related to twin-to-twin delivery time interval in minutes

Figure 2 shows the correlation between arterial umbilical pH and twin-to-twin interval time.

**Fig. 3:**
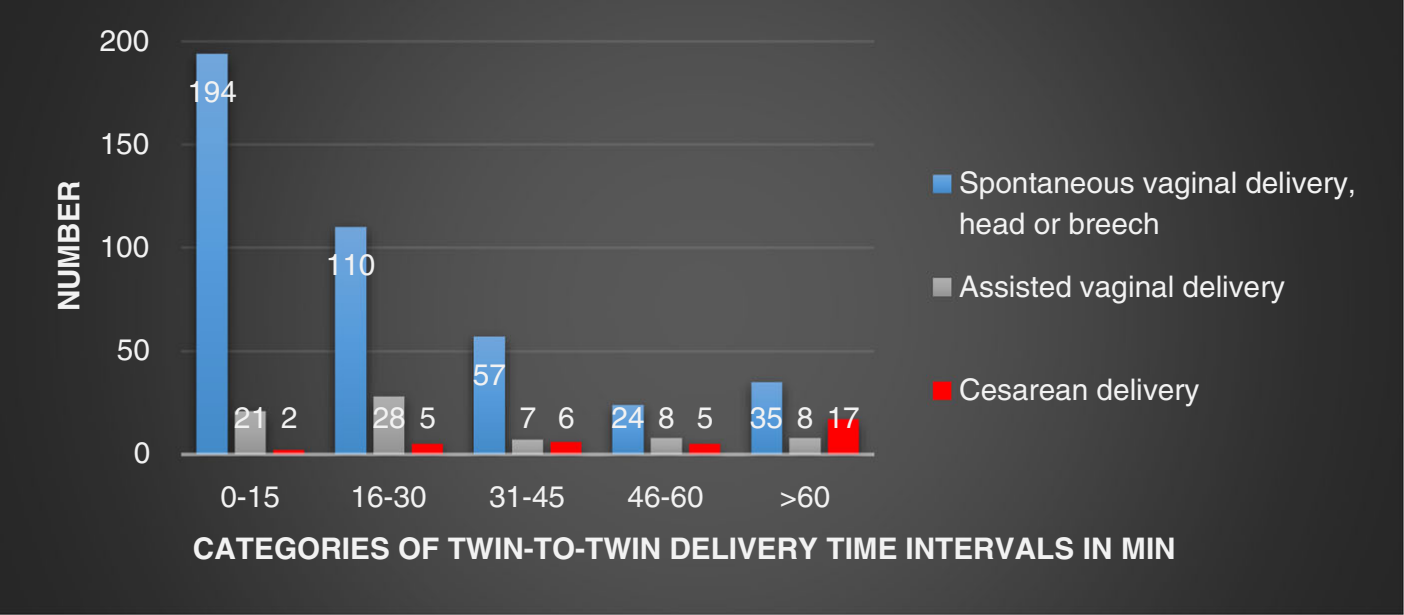
Mode of delivery of the second twin according to twin-to-twin delivery time interval in minutes

Most of the CS to deliver the second twin occurred in the group with a twin-to-twin time interval > 30 min 28/35 vs. 7/35 ≤ 30 min, *p* < 0.0001). The indications for CS to deliver the second twin were fetal distress (*n* = 15), malpresentation (*n* = 9), uterine inertia (*n* = 5), cord prolapse (*n* = 3), placental separation (*n* = 1), failed internal version (*n* = 1) and failed external version (*n* = 1). The mode of delivery for the second twin in relation to twin-to-twin time interval is shown in Fig. 3.

## Discussion

This study reaffirms previous evidence that the second twin is at greater risk of metabolic acidosis and neonatal morbidity than the first twin [3, 6, 12, 18]. We were able to confirm that there is an association, but not a clear causality, with the twin-to-twin time interval [11, 12]. Only 14 of the second twins had a composite primary outcome but they had significantly longer median twin-to-twin time intervals than second twins without a composite primary outcome. Second twins with a twin-to-twin time interval of more than 30 min had higher rates of primary composite outcomes than second twins born within 30 min. No differences were seen in composite secondary outcomes that related to twin-to-twin time intervals.

Previous studies have come to varying results on the possible impact of twin-to-twin time interval on neonatal outcome for the second twin [9, 11, 13]. Most studies imply that considering the fact that umbilical cord pH decreases with longer duration of the delivery of the second twin, it is important to apply active management to keep the twin-to-twin time interval as short as possible, ideally below 30 min. Leung found that pH deteriorates faster in the second twin than in the first twin [19], and recommend a fast delivery of the second twin. In this study we found a significantly higher rate of metabolic acidosis and lower mean arterial pH in second twins born after a twin-to-twin time interval of 30 min than in second twins born within 30 min. However, there was no difference in Apgar score, the differences in pH were small and most of the second twins with metabolic acidosis recovered quickly. The two cases of perinatal mortality occurred in second twins born within 30 min. Furthermore, there was no difference in neonatal morbidity, and admission to NICU was not associated with twin-to-twin time interval.

Although the small difference in pH-levels in umbilical blood gas (7.23 vs. 7.20) for second twins delivered within or after 30 min may not have a clinical impact, other findings may justify an upper time limit on the interval between twin-to-twin deliveries. In this study there was a combined vaginal-cesarean delivery in 35 cases (6.6%), a rate consistent with other findings [20]. Cesarean delivery of the second twin is considered to be the least desirable mode of delivery and should be avoided [4, 6] due to possible complications for both mother and child. Studies have shown a worsened outcome, with increased physical and psychological maternal morbidity [18, 21] as well as higher neonatal morbidity [4, 6]. Twenty eight of these 35 combined vaginal-cesarean deliveries occurred in the twin-to-twin time interval of more than 30 min, 17 in the interval of more than 60 min. This is in agreement with previous studies that have found a six-fold increased risk of combined vaginal-cesarean delivery after 30 min and an eight-fold increased risk after 60 min [4]. Our study does not show any differences in primary and secondary neonatal outcome related to CS. However, maternal physical and psychological morbidity was not analyzed.

The fact that most combined deliveries occurred in the group where the twin-to-twin delivery time interval was more than 30 min, is in itself not surprising. If the second twin is not spontaneously delivered, the risk of assisted delivery increases over time. Previous studies have found that combined vaginal-cesarean twin delivery can be avoided with active management in the second stage of delivery of the second twin [22]. Active management is generally considered to be internal podalic version (IPV) followed by breech extraction of the non-vertex and the unengaged vertex second twin. Previous findings suggest that IPV may be more successful than external version when it comes to vaginal delivery, and with better neonatal outcome [1, 22–24]. External version has been found to be associated with complications such as fetal distress, cord prolapse and compound presentations [25]. However, this maneuver was only performed in 10 cases, of which nine were successful. Active management is not praxis at our unit and unfortunately the IPV maneuver is therefore seldom taught to junior obstetricians. A decreasing rate of IPV and breech extraction, and an increased rate of CS, has also been seen in recent years in several other countries around the world [24]. In the Danish register study a better neonatal outcome was seen for the second twin after IPV and extraction than after a CS [24]. If an upper time limit is to be advocated, the management of the delivery of the second twin would need to be more active than what is recommended at our unit today. This may lead to an increase in use of oxytocin, instrumental deliveries (VE/forceps), breech extraction of a second non vertex twin and IPV and breech extraction of a second vertex non-engaged twin. Without adequate knowledge of, and training in, active management the clinical outcome of such a change in strategy is unknown.

Our unit is able to monitor both twins simultaneously and continuously. We also have 24-h in-house pediatric and anesthesia coverage and immediate availability for performing emergency CS. This may partly explain the few primary outcomes. Because of this the results of this study cannot be extrapolated to other units with other preconditions. It is important for every unit delivering twins to look at their results and from this stipulate guidelines that are in accordance with their preconditions. Factors such as the level of experience of the obstetrician in charge at the delivery, the skill of intrauterine manipulation and the wish of the women giving birth, may all influence the twin-to-twin delivery time interval. Results from the Twin Birth Study showed that the maternal preference is for vaginal birth, therefore skills need to be maintained [26].

The limitations of the study are, as in all retrospective studies, that we have no control over available information. A rather large number of the twins had missing pH values (23% in the first twin and 19.2% in the second twin). However, analysis was made of complete data on more than 400 twins in each group. Despite a relatively large sample size there were few primary outcomes and regression analysis could not be performed for all potential confounders. The results are to be analyzed in the light of the relatively high gestational age of the sample. Twin gestations delivered before 32 + 0 weeks of gestation are complicated per see and excluding them may result in a selected sample. Including them might on the other hand result in morbidity outcomes that are not related to twin-to-twin delivery interval but prematurity in itself. There may also be other important confounders that we did not have information on. A randomized controlled trial with different twin-to-twin time intervals would be the optimal study design, but would probably be impossible to perform. Further, in this study we have not addressed the impact on maternal morbidity as postpartum hemorrhage and length of hospital stay etc.

## Conclusions

An association, but not necessarily causality, between lengths of twin-to-twin time intervals and primary outcome in second twins, was found. Based on the findings of this study an upper time limit for twin-to-twin time intervals may be justified, due to the increased rate of metabolic acidosis and combined vaginal-cesarean deliveries associated with prolonged intervals between deliveries. However, the optimal twin-to-twin time interval needs further study before any recommendation can be made. Furthermore, active second stage management of the second twin needs an obstetrician with skills in the requisite maneuvers, so adequate training of the next generation of obstetricians is therefore necessary.

## Abbreviations

BE: Base excess
BMI: Body mass index
CPAP: Continuous positive air pressure
CS: Cesarean section
HIE: Hypoxic ischemic encephalopathy
IPV: Internal podalic version
IRDS: Irregular respiratory distress syndrome
IVH: Intraventricular hemorrhage
LGA: Large for gestational
MRI: Magnetic resonance imaging
NEC: Necrotizing enterocolitis
NICU: Neonatal intensive care unit
OR: Odds ratio
SD: Standard deviation
SGA: Small for gestational age
SNQ: Swedish neonatal quality register
TTN: Transient tachypnea of the newborn
TTTS: Twin-twin transfusion syndrome
